# The matricellular protein CCN5 inhibits fibrotic deformation of retinal pigment epithelium

**DOI:** 10.1371/journal.pone.0208897

**Published:** 2018-12-20

**Authors:** Aeri Yoon, Sora Im, Juyeon Lee, Daeho Park, Dong Hyun Jo, Jin Hyoung Kim, Jeong Hun Kim, Woo Jin Park

**Affiliations:** 1 College of Life Sciences, Gwangju Institute of Science and Technology, Cheomdangwagi-ro, Buk-gu, Gwangju, Republic of Korea; 2 Department of Biomedical Sciences, Seoul National University College of Medicine, Daehak-ro, Jongno-gu, Seoul, Republic of Korea; 3 Fight Against Angiogenesis-Related Blindness Laboratory, Biomedical Research Institute, Seoul National University Hospital, Daehak-ro, Jongno-gu, Seoul, Republic of Korea; Duke University, UNITED STATES

## Abstract

Retinal pigment epithelium (RPE) plays an essential role in maintaining retinal function, and its defect is thought to be critically implicated in various ocular disorders. This study demonstrated that the matricellular protein CCN5 was down-regulated in ARPE-19 cells treated with the pro-fibrotic agent transforming growth factor (TGF)-β. A recombinant adenovirus expressing CCN5 (AdCCN5) was used to restore the level of CCN5 in these cells. AdCCN5 prevented TGF-β-induced fibrotic changes, including disruption of tight junctions, up-regulation of mesenchymal marker proteins, and down-regulation of epithelial marker proteins. In addition, AdCCN5 prevented TGF-β-induced functional defects, including increased migratory activity and reduced phagocytic activity. Notably, AdCCN5 reversed morphological and functional defects pre-established by TGF-β prior to viral infection. The CCN5 level was down-regulated in RPE of 18-month-old *Ccl2*^-/-^ mice, which exhibited retinal defects. Restoration of the CCN5 level via intravitreal injection of a recombinant adeno-associated virus expressing CCN5 (AAV9-CCN5) normalized the altered expression of mesenchymal, epithelial, and functional marker proteins, as assessed by western blotting and immunohistochemistry. Taken together, these data suggest that down-regulation of CCN5 is associated with fibrotic deformation of RPE under pathological conditions and that restoration of the CCN5 level effectively promotes recovery of deformed RPE.

## Introduction

Retinal pigment epithelium (RPE) is a highly polarized monolayer of cuboidal cells located between the neural retina and choroid that plays a pivotal role in maintaining retinal function [1]. Normal RPE has a mature epithelial phenotype with morphologically and functionally asymmetric structures [2]. Deformation of RPE cells is regarded as a primary event leading to progressive fibrotic diseases of the eye, including proliferative vitreoretinopathy (PVR), diabetic retinopathy (DR), and age-related macular degeneration (AMD) [3]. In pathological conditions, RPE cells undergo fibrotic deformation characterized by loss of cellular integrity and perturbation of functions, such as phagocytosis and directional transcytosis [4–6]. This pathological process is thought to be triggered by a variety of intravitreal cytokines, among which transforming growth factor (TGF)-β is the best characterized [7]. *In vitro* experiments demonstrated that TGF-β contributes to an aberrant wound response in RPE [8] and that its level significantly correlates with excessive deposition of extracellular matrix components in RPE [9]. In addition, the TGF-β level is significantly elevated in patients with PVR, DR, and AMD [10–13]. These observations suggest that TGF-β-mediated fibrotic deformation of RPE plays a role in various retinal pathologies.

CCN5 (also known as Wnt-1-induced signaling protein-2; WISP2) is a member of the CCN family of matricellular proteins (CCN1–6) [14] and has been implicated in a variety of human diseases [15]. The role of CCN5 in human pathology is mainly attributed to its function in proliferation and mobilization of breast cancer and pancreatic adenocarcinoma cells [16, 17] and proliferation of vascular smooth muscle cells [18]. We previously showed that CCN5 prevents pressure overload-induced cardiac fibrosis partly by inhibiting the TGF-β-SMAD signaling pathway in mice [19]. At the cellular level, CCN5 inhibits endothelial-mesenchymal transition and transdifferentiation of fibroblasts into myofibroblasts in the heart, the two most critical processes underlying cardiac fibrosis. We further showed that CCN5 can reverse pre-established cardiac fibrosis by inducing apoptosis specifically in myofibroblasts, but not in fibroblasts or myocytes [20]. CCN5 also induces phenotypic reversion in some populations of myofibroblasts (unpublished observations).

The present study demonstrated that CCN5 was markedly down-regulated in RPE cells treated with TGF-β. Restoration of the CCN5 level via infection of a recombinant adenovirus harboring CCN5 (AdCCN5) prevented and reversed TGF-β-mediated fibrotic deformation of RPE cells. CCN5 elicited similar beneficial effects in aged *Ccl2*^-/-^ mice, which exhibited RPE deformation. Collectively, this study suggests that CCN5 is a useful target for the treatment of various ocular diseases that are primarily caused or accompanied by fibrotic deformation of RPE.

## Materials and methods

### Animals

All animal experimental methods and protocols in this study were approved by the Institutional Animal Care and Use Committee of the School of Life Sciences, Gwangju Institute of Science and Technology, and carried out in accordance with their approved guidelines (IACUC GIST-2015-24). *Ccl2*^-/-^ mice (Jackson Laboratories, USA) generated as described previously [21, 22] were backcrossed ten times with C57BL/6 wild type (WT) mice (Damul Science, Korea). AAV9-VLP or AAV9-CCN5 was delivered into 18-month-old *Ccl2*^-/-^ mice for 12 weeks. Age-matched WT littermates administered AAV9-VLP were used as a control. Surgery was performed under deeply anesthetized via intraperitoneally injecting a mixture of Zoletil 50 (Virbac, France) and Rompun (Bayer Korea, Korea) at a ratio of 3:1 (1 ml/kg), and all efforts were made to minimize suffering. After surgical procedure, mice were monitored in every other day to check whether any adverse events were occurred. As far as we have observed, we have not found any other adverse clinical signs or gross abnormalities in other organ systems to date. At the end of experiments, we administered CO_2_ inhalation for euthanasia and the eyes were enucleated immediately.

### Intravitreal injection

AAV9-CCN5 was intravitreally injected using a Nanofil syringe with a 33G blunt needle (World Precision Instruments Inc., USA) underneath an operating microscope (Leica Microsystems Ltd., Germany). The same concentration of AAV9-VLP was injected into control mice.

### Cells and cell culture

ARPE-19 cells were obtained from the American Type Culture Collection (ATCC, USA) and cultured in a 1:1 mixture of Dulbecco’s modified Eagle’s medium and Ham’s F12 Nutrient Mixture (DMEM/F12; Welgene, Korea) containing 10% fetal bovine serum (FBS; HyClone, USA), 1 mM glutamine, and 100 U/ml penicillin/streptomycin at 37°C in a humidified atmosphere containing 5% CO_2_. The medium was changed every 2 days. Confluent cells were dissociated with 0.25% trypsin/0.02% ethylenediaminetetraacetic acid solution (Gibco, USA). Fully confluent ARPE-19 cells were serum-starved for 24 hours, and then the medium was replaced by that containing AdCCN5 at a MOI of 50 or 10 ng/ml TGF-β2 (PeproTech Korea, Korea) prior to TGF-β treatment or AdCCN5 infection, respectively.

### AAV and adenovirus vectors

An AAV serotype 9 construct harboring the human CCN5 gene under the control of the CMV promoter was produced and purified by Virovek (USA). Full-length mouse CCN5 cDNA tagged with hemagglutinin at the amino-terminal was generated and purified as previously described [19].

### Western blot analysis

ARPE-19 cells were washed with phosphate-buffered saline (PBS) and harvested by scraping. Cell pellets were suspended in cold RIPA lysis buffer (1% NP-40, 50 mM Tris-HCl [pH 7.4], 150 mM NaCl, and 10 mM NaF) supplemented with a mammalian cell protease inhibitor cocktail (Roche, Germany) and sonicated at an amplitude of 50% for 5 minutes with a cycle of 2 seconds on/1 second off. To extract proteins from the RPE/choroid/sclera complex of *Ccl2*^-/-^ mice, enucleated eyes without the neural retina were suspended in cold RIPA lysis buffer containing protease inhibitor and sonicated at an amplitude of 50% for 10 seconds with a cycle of 2 seconds on/2 seconds off using a probe sonicator with a 1/8 inch microtip. Lysates were centrifuged at 13,000 rpm for 20 minutes, and the protein concentration of the supernatant was determined using a bicinchoninic acid protein assay kit (Thermo Fisher Scientific, USA). Equal amounts of protein samples were mixed with 5× SDS sample buffer, subjected to SDS-PAGE, and transferred to polyvinylidene difluoride membranes (Millipore, USA). Membranes were blocked with Tris-buffered saline-Tween 20 containing 5% non-fat dry milk powder for 1 hour, incubated overnight at 4°C with specific primary antibodies (S1 Table), and then labeled with the appropriate horseradish peroxidase-conjugated secondary antibodies (Invitrogen, USA). Immune complexes were visualized using an enhanced chemiluminescence reagent (Amersham, UK) and an ImageQuant LAS 4000 Mini imager (GE Healthcare, UK). The intensity of each protein band was quantified using NIH ImageJ software (NIH, USA).

### Immunofluorescence

Cells cultured on coverslips in a 12-well culture plate were fixed with 4% paraformaldehyde for 10 minutes and permeabilized with 0.2% Triton X-100 prepared in PBS for 10 minutes. After blocking for 1 hour with 5% bovine serum albumin prepared in PBS at room temperature, cells were incubated with primary antibodies overnight at 4°C and then with FITC- or Alexa Fluor 594-conjugated secondary antibodies (Invitrogen, USA) for 1 hour at room temperature. Cells were stained with Texas Red-conjugated phalloidin to visualize F-actin. Nuclei were stained with Hoechst 33342 (Invitrogen, USA). Samples were analyzed underneath a microscope equipped with a 20× objective lens and epifluorescence filters (Zeiss, Germany). For flat mount of RPE/choroid/sclera complex, enucleated eyes were dissected to remove neural retina and gently flat mounted and fixed in methanol for 15 minutes at -20°C. After washing with PBS, RPE complex were incubated in 0.2% Triton X-100 for 2 hours at 37°C. After blocking for an hour at 37°C, the RPE complex were incubated at 4°C overnight with primary antibody. After washing with PBS, the RPE complex were incubated for 2 hours at 37°C with secondary antibodies. After washing with PBS, flat mounts were counterstained with 10 mg/mL of 4, 6-diamidino-2-phenolindole (DAPI; Sigma-Aldrich, USA). After washing with PBS, the RPE complex were mounted with aqueous mounting medium and observed under fluorescence microscope. Information about the primary antibodies is provided in S1 Table.

### RNA extraction and quantitative Real-time PCR analysis

Total RNA was isolated from treated cells using Trizol reagent (Invitrogen, USA) according to the manufacturer’s instruction. cDNA was synthesized with a reverse transcription kit (Promega, USA) and amplified via Real-time PCR using the SYBR green (Takara, Japan). The specificity of the amplification reactions was confirmed by melting curve analysis. Samples were assayed in triplicate or quadruplicate. RNA was quantified using the ΔΔCt method of relative quantification (RQ) and the transcript levels were normalized to the endogenous control 18s rRNA. Information of the primer sequence is shown in S2 Table.

### Collagen gel contraction assay

Collagen gel contraction assays were performed as previously described [23]. In brief, 1.2 mg/ml collagen solution (Invitrogen, USA), 10× DMEM/F12, 0.5N NaOH and ARPE-19 cells (1 × 10^5^ cells per well) were mixed and added to a 24-well culture plate. After incubation for 1 hour at 37°C to allow collagen polymerization, the gelatinous suspension was detached from the well edges using a sterile pipette tip. The collagen gel was allowed to float in serum free DMEM/F12 media containing TGF-β and AdLacZ or AdCCN5. ImageJ software was used to calculate the extent of collagen gel contraction.

### *In vitro* cell scratch assay

ARPE-19 cells were plated on coverslips in a 12-well culture plate in DMEM/F12 medium. When the monolayer was fully confluent, the medium was replaced by serum-free medium and cells were cultured for 12 hours. Following starvation, cells were infected with AdCCN5 for 24 hours prior to treatment with 10 ng/ml TGF-β for 48 hours in the prevention model. Cells were treated with AdCCN5 and TGF-β in the opposite order in the reversion model. The monolayer was scratched by gently and slowly dragging a pipette tip across the center of the well. After 24 hours, cells were stained with DAPI and closure of the gap via cell migration was monitored by microscopy. The gap width was measured using NIH ImageJ software.

### Phagocytosis assay

ARPE-19 cells were seeded into a 24-well culture plate at a density of 5 × 10^4^ cells per well and treated with the indicated virus (AdLacZ or AdCCN5) and TGF-β. The effect of photodynamic stress on phagocytic activity of the cells was determined by challenging control and photodynamically treated cells with TAMRA-stained apoptotic thymocytes or 1 mg/ml pHrodo Red BioParticles conjugates (Invitrogen, USA) in 5% CO_2_ at 37°C for 6 and 4 hours, respectively. To generate TAMRA-labeled apoptotic thymocytes, a thymus was acquired from a 5–6-week-old C57BL/6 mouse and gently dissociated using a 5 ml syringe piston and a cell strainer to separate single thymocytes. Thymocytes were stained with 50 μM TAMRA-SE (Invitrogen, USA) prepared in HBSS in a CO_2_ incubator at 37°C for 30 minutes, de-stained in RPMI containing 10% FBS and 1% penicillin/ streptomycin/ glutamine in 5% CO_2_ at 37°C for 20 minutes, and washed once with complete RPMI. Apoptosis of thymocytes was induced by treatment with 50 μM dexamethasone (Calbiochem, Germany) in a CO_2_ incubator at 37°C for 4 hours. Thereafter, cells were washed thrice with complete RPMI, and 20 × 10^5^ apoptotic thymocytes were resuspended in 300 μl of phagocyte culture medium. Apoptotic thymocytes were incubated with treated ARPE-19 cells in 5% CO_2_ at 37°C. Thereafter, phagocytes were washed five times with ice-cold PBS, trypsinized, suspended in complete culture medium, and analyzed using a FACSCantoII flow cytometer (BD, USA). ARPE-19 cells were gated according to the forward scatter/side scatter plot to distinguish non-ingested apoptotic thymocytes or BioParticles, cell fragments, and other debris from single cells. An appropriate negative control (cells without apoptotic thymocytes or BioParticles) was used in each experiment. After using the marker M1 to gate positive cells, the percentage of fluorescence-positive events from 20,000 live cells per sample was calculated. Data were analyzed using FlowJo software.

### Statistics

Quantitative functional and gene expression assays were performed at least three times. Mean averages ± S.D. were calculated. Two groups were compared using the two-tailed Student’s t-test. Statistical significance was indicated by a single asterisk (*, p < 0.05) or a double asterisk (**, p < 0.01).

## Results

### CCN5 prevents TGF-β-induced fibrotic deformation of ARPE-19 cells

**Fig 1:**
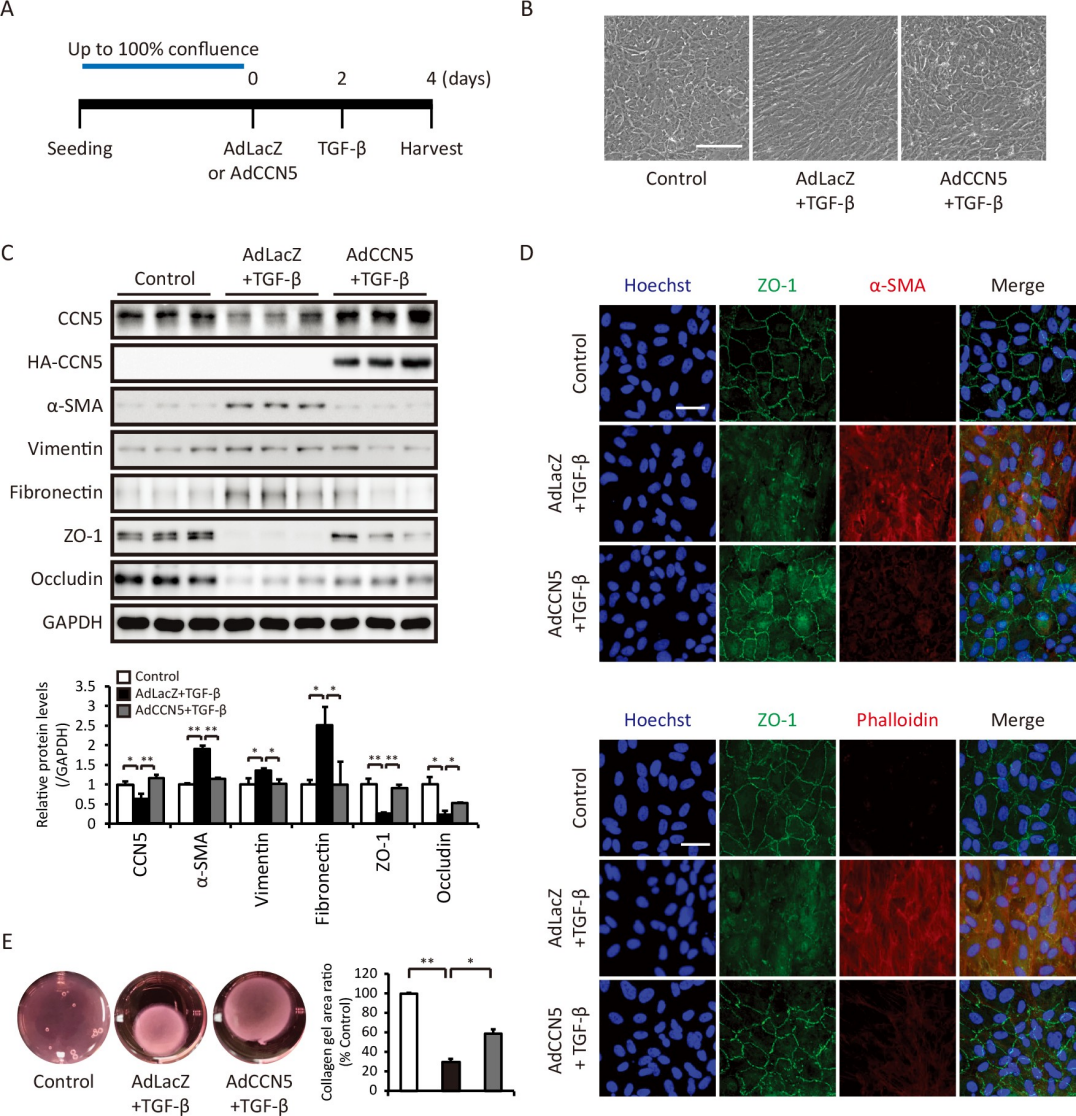
CCN5 prevents TGF-β-induced fibroblast-like phenotypic transformation of ARPE-19 cells. (A) ARPE-19 cells were grown until 100% confluent and cultured in the presence of TGF-β and AdLacZ or AdCCN5. (B) Bright field microscopy images showing the change in the morphology of ARPE-19 cells from a monolayer of cuboidal cells to mesenchymal-like cells upon treatment with 10 ng/ml TGF-β. This change was not observed in cells pretreated with AdCCN5. Original magnification: 100×. Scale bar: 200 μm. (C) Total cell lysates obtained under the same conditions as in A were immunoblotted with antibodies against CCN5, α-SMA, vimentin, fibronectin, ZO-1, and occludin. GAPDH was used as a loading control. Quantified protein levels were normalized against the loading control (bottom). Error bars = S.D. *p < 0.05, **p < 0.01. (D) Immunofluorescence images of ARPE-19 cells stained for ZO-1 and α-SMA. Nuclei were counterstained with Hoechst. Images are representative of at least three independent experiments. Original magnification: 200×. Scale bar: 50 μm. (E) Collagen gel lattices were generated and cultured under the same conditions as shown in A. Areas of collagen gel contraction were measured after 48 hours. Representative images of collagen gel lattices are shown (left), and areas of gel contraction were quantified (right) (n = 5). Error bars = S.D. *p < 0.05, **p < 0.01.

ARPE-19 is a cell line spontaneously derived from human RPE. Post-confluent ARPE-19 cells form a cobblestone-like monolayer and express typical epithelial marker proteins. ARPE-19 cells underwent phenotypic transformation and acquired an elongated and fibroblastic morphology upon treatment with 5 or 10 ng/ml TGF-β for 48 hours (S1A Fig). Notably, TGF-β treatment significantly decreased the CCN5 protein level. In parallel, levels of mesenchymal marker proteins, including α smooth muscle actin (α-SMA), vimentin, fibronectin, and type I collagen, were elevated, whereas levels of epithelial marker proteins, including zonula occludens (ZO)-1 and occludin, were significantly reduced (S1B Fig). Furthermore, immunocytochemistry revealed that the levels of α-SMA and filamentous (f-) actin were elevated upon treatment with TGF-β (S2 Fig). This confirmed that TGF-β treatment induced fibrosis-like phenotypic transformation of ARPE-19 cells. We thus treated ARPE-19 cells with 10 ng/ml TGF-β for 48 hours to induce fibrotic deformation in all subsequent experiments. Cells were infected with the recombinant adenovirus AdCCN5, which expressed CCN5 under the control of the CMV promoter, or with the control virus AdLacZ. At 2 days post-infection, cells were treated with TGF-β for an additional 2 days (Fig 1A). AdCCN5 markedly prevented the morphological changes induced by TGF-β (Fig 1B). Western blotting showed that AdCCN5 prevented the TGF-β-induced increases and decreases in levels of mesenchymal and epithelial marker proteins, respectively (Fig 1C). These changes were further confirmed by qRT-PCR analyses (S3 Fig). Immunocytochemical staining of ZO-1 revealed that TGF-β markedly disrupted tight junctions and that this was prevented by AdCCN5. AdCCN5 also prevented TGF-β-mediated induction of α-SMA and appearance of f-actin, as detected by immunostaining and phalloidin staining (Fig 1D). Enhanced contractility due to increased α-SMA expression is a hallmark of fibrotic transformation of epithelial cells. The collagen gel contraction assay demonstrated that TGF-β increased contractility and that this was significantly inhibited by AdCCN5 (Fig 1E). These results indicate that CCN5 prevents TGF-β-induced fibrotic deformation of ARPE-19 cells.

### CCN5 prevents TGF-β-induced functional deterioration of ARPE-19 cells

**Fig 2:**
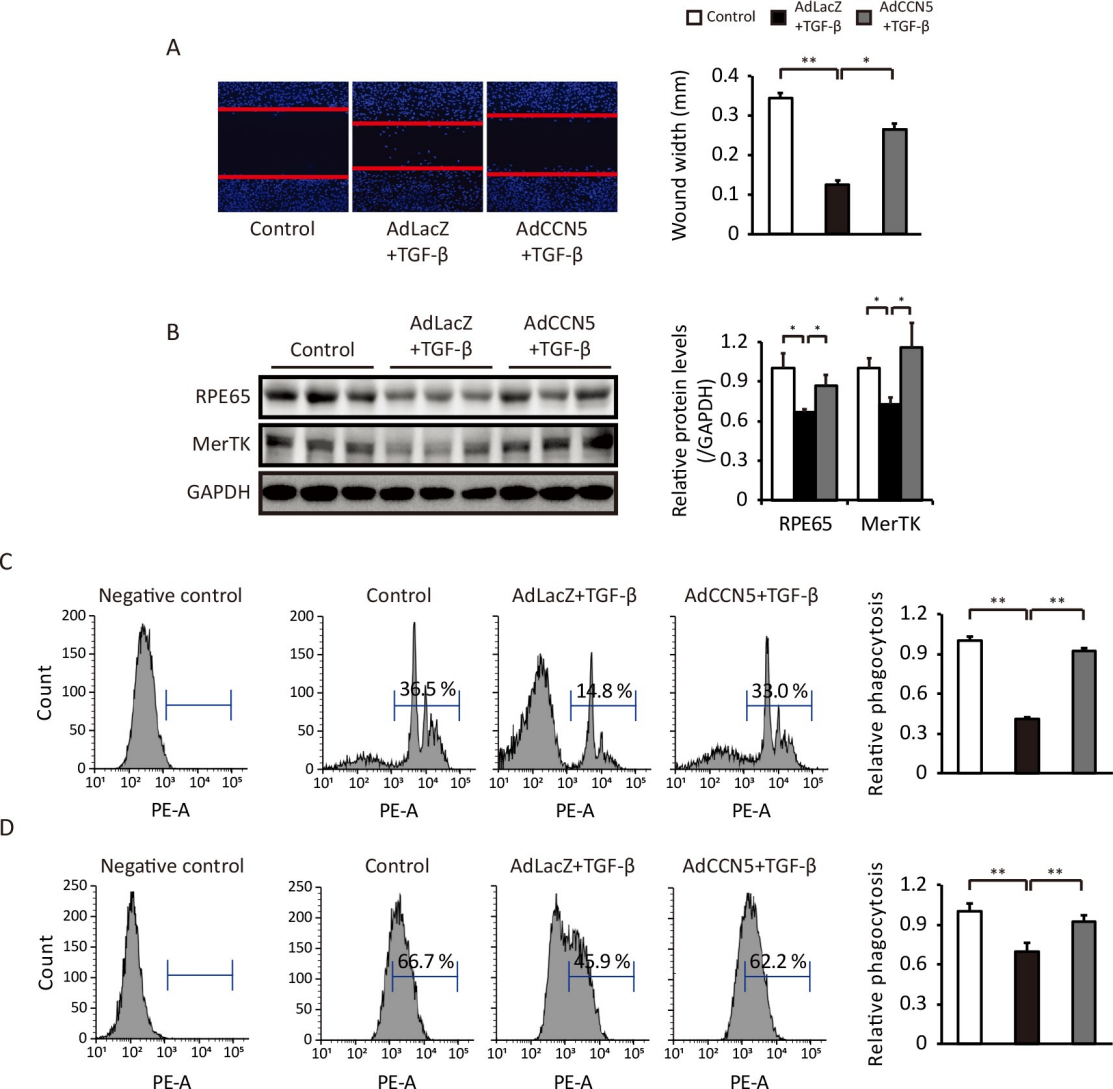
CCN5 prevents TGF-β-induced functional deterioration of ARPE-19 cells. (A) ARPE-19 cells were cultured in the presence of TGF-β and AdLacZ or AdCCN5 for 48 hours, scratched, fixed, and stained with Hoechst. Representative images are shown (left), and migration distances were quantified (right) (n = 3). Error bars = S.D. *p < 0.05, **p < 0.01. (B) Expression of the RPE function-related proteins RPE65 and MerTK was analyzed by western blotting under the same conditions as in A. Error bars = S.D. *p < 0.05. (C) Phagocytic function of ARPE-19 cells was analyzed using pHrodo BioParticles conjugates under the same conditions as in A. TGF-β reduced phagocytic activity, and this was inhibited by pretreatment with AdCCN5. Phagocytosis was quantified by flow cytometry. The relative fluorescence intensity of internalized pHrodo BioParticles was quantified (right) (n = 9). Error bars = S.D. **p < 0.01. (D) TAMRA-labeled apoptotic thymocytes were added to ARPE-19 cells under the same conditions as shown in A and examined by flow cytometry. The relative fluorescence intensity of internalized apoptotic thymocytes was quantified (right) (n = 6). Error bars = S.D. **p < 0.01.

TGF-β triggers functional deterioration, as well as morphological transformation, of ARPE-19 cells. ARPE-19 cells were infected with AdCCN5 or AdLacZ for 2 days and then treated with TGF-β for an additional 2 days. Thereafter, a scratch was made across the monolayer and cells were examined underneath a microscope after 24 hours. TGF-β enhanced the migratory activity of ARPE-19 cells, and this was significantly inhibited by AdCCN5 (Fig 2A). The RPE65 enzyme converts all-trans retinal into 11-cis retinal during the vision cycle and is essential for normal vision, while MerTK plays a critical role in phagocytosis by RPE cells. Western blotting showed that the protein levels of RPE65 and MerTK were significantly reduced by TGF-β and that this was inhibited by AdCCN5 (Fig 2B). We used two pH-sensitive fluorophore-labeled materials, pHrodo Red BioParticles and tetramethyl-6-carboxyrhodamine (TAMRA)-labeled apoptotic thymocytes, to analyze the phagocytic function of RPE cells. These fluorophores are activated when the materials are engulfed by acidic phagosomes. RPE cells were incubated with these materials and analyzed by flow cytometry. TGF-β markedly reduced the phagocytic activity of RPE cells, and this was significantly attenuated by AdCCN5 (Fig 2C and 2D). These data indicate that CCN5 prevents TGF-β-induced functional deterioration of ARPE-19 cells.

### CCN5 reverses TGF-β-induced fibrotic deformation of ARPE-19 cells

**Fig 3:**
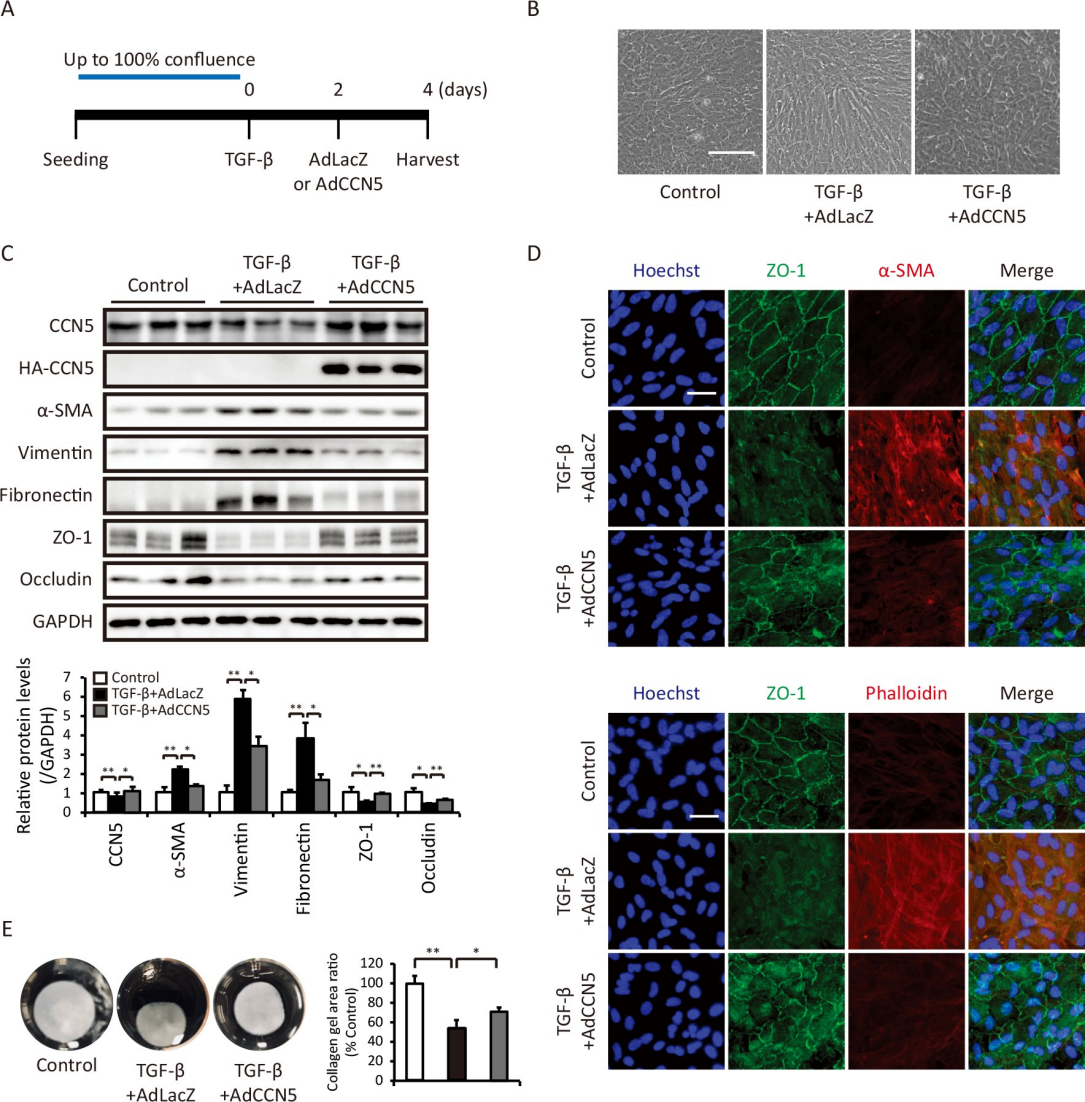
CCN5 reverses TGF-β-induced fibrotic deformation of ARPE-19 cells. (A) Fully confluent ARPE-19 cells were serum-starved for 24 hours, treated with TGF-β, and then infected with AdLacZ or AdCCN5. (B) Bright field microscopy images showing the change in the morphology of ARPE-19 cells from an organized epithelial cobblestone-like monolayer to fibroblast-like cells upon treatment with TGF-β in the absence of AdCCN5. Original magnification: 100×. Scale bar: 200 μm. (C) Total cell lysates prepared under the same conditions as shown in A were immunoblotted with antibodies against CCN5, α-SMA, vimentin, fibronectin, ZO-1, and occludin. GAPDH was used as a loading control. Quantified protein levels were normalized against the loading control (left bottom). Error bars = S.D. *p < 0.05, **p < 0.01. (D) Immunofluorescence images of ARPE-19 cells stained for ZO-1 and α-SMA. Nuclei were counterstained with Hoechst. Images are representative of at least three independent experiments. Original magnification: 200×. Scale bar: 50 μm. (E) Collagen gel lattices were generated and cultured under the same conditions as shown in A. Representative images of collagen gel lattices are shown (left), and areas of gel contraction were quantified (right) (n = 3). Error bars = S.D. *p < 0.05, **p < 0.01.

We next examined whether CCN5 could reverse pre-established TGF-β-induced fibrotic deformation of ARPE-19 cells. To this end, confluent ARPE-19 cells were treated with TGF-β for 2 days and then infected with AdCCN5 or AdLacZ (Fig 3A). Microscopic analysis showed that AdCCN5 markedly reversed TGF-β-induced morphological transformation of ARPE-19 cells (Fig 3B). Western blotting demonstrated that AdCCN5 significantly normalized the TGF-β-induced increases in levels of mesenchymal marker proteins (α-SMA, vimentin, and fibronectin) and decreases in levels of epithelial marker proteins (ZO-1 and occludin) (Fig 3C). Immunostaining with an anti-ZO-1 antibody revealed that tight junctions disrupted by TGF-β were markedly restored by AdCCN5. In addition, AdCCN5 normalized the α-SMA level, which was increased by TGF-β. Phalloidin staining revealed that AdCCN5 completely eliminated the TGF-β-induced formation of f-actin (Fig 3D). The collagen gel contraction assay also demonstrated that TGF-β increased contractility and that this was restored by AdCCN5 (Fig 3E). These results show that CCN5 can reverse TGF-β-induced fibrotic deformation of ARPE-19 cells.

### CCN5 reverses TGF-β-induced functional deterioration of ARPE-19 cells

**Fig 4:**
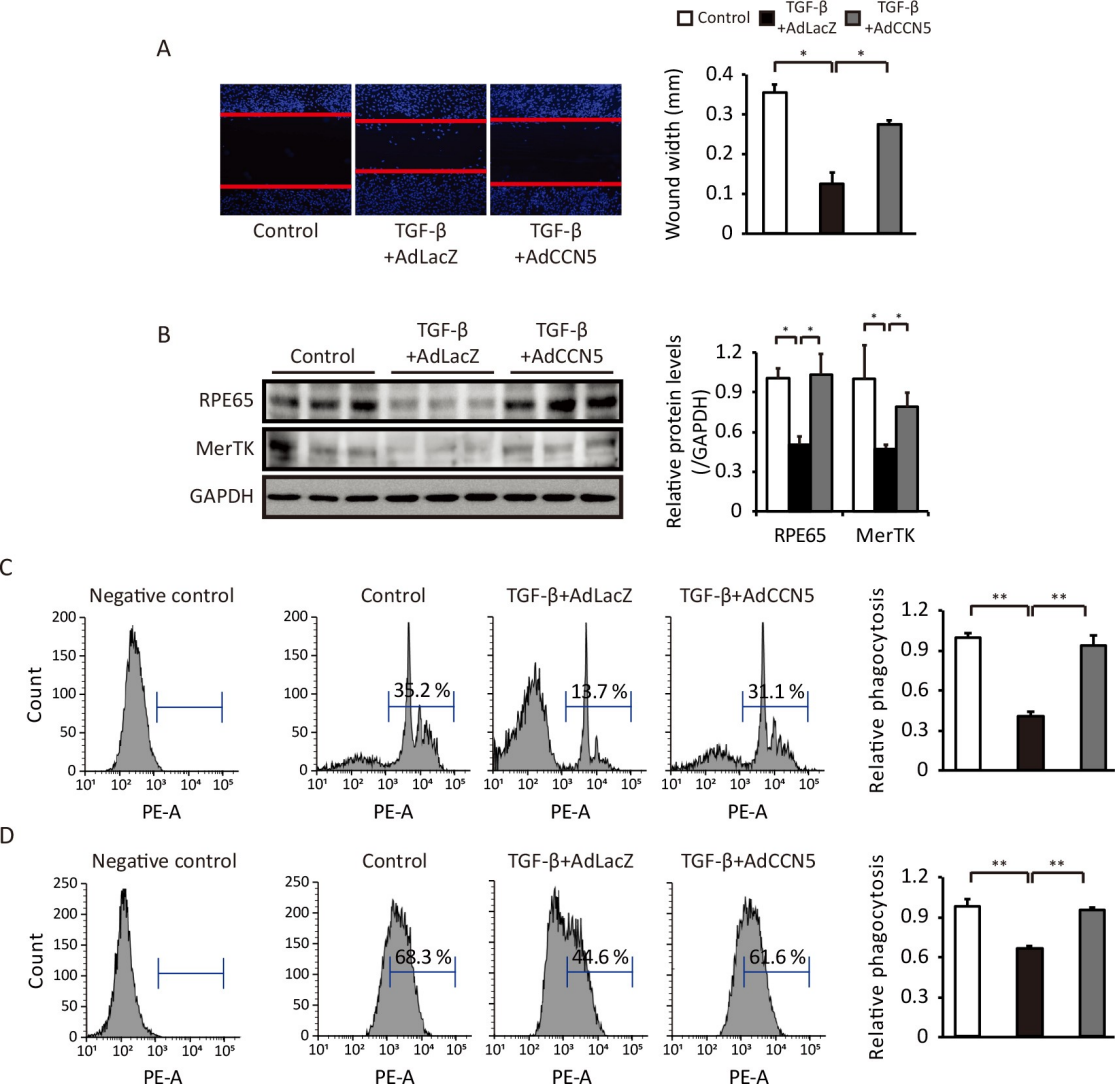
CCN5 reverses TGF-β-induced functional deterioration of ARPE-19 cells. (A) ARPE-19 cells were treated with TGF-β and then infected with AdLacZ or AdCCN5. After 48 hours, cells were scratched, fixed, and stained with Hoechst. Representative images are shown (left), and migration distances were quantified (right) (n = 3). Error bars = S.D. *p < 0.05. (B) Expression of the RPE function-related proteins RPE65 and MerTK was determined by western blotting under the same conditions as in A. Error bars = S.D. *p < 0.05. (C) Phagocytic function of ARPE-19 cells was analyzed using pHrodo BioParticles conjugates under the same conditions as shown in A. TGF-β-induced phagocytic dysfunction was recovered in the presence of AdCCN5. Phagocytosis of ARPE-19 cells was quantified by flow cytometry. The relative fluorescence intensity of internalized pHrodo BioParticles was quantified (right) (n = 9). Error bars = S.D. **p < 0.01. (D) TAMRA-labeled apoptotic thymocytes were added to ARPE-19 cells under the same conditions as shown in A and analyzed by flow cytometry. Relative fluorescence intensity of internalized apoptotic thymocytes was quantified (right) (n = 6). Error bars = S.D. **p < 0.01.

ARPE-19 cells treated as described in the previous section were subjected to functional analyses. TGF-β-induced migration of ARPE-19 cells was reduced by AdCCN5 to a level comparable to that in the control group (Fig 4A). AdCCN5 significantly normalized the TGF-β-induced decreases in the protein levels of RPE65 and MerTK (Fig 4B). Flow cytometric analyses revealed that TGF-β-induced suppression of phagocytic activity was significantly recovered by AdCCN5 (Fig 4C and 4D). These results indicate that CCN5 can reverse TGF-β-induced functional defects of ARPE-19 cells.

### CCN5 reverses phenotypic deformation of RPE *in vivo*

**Fig 5:**
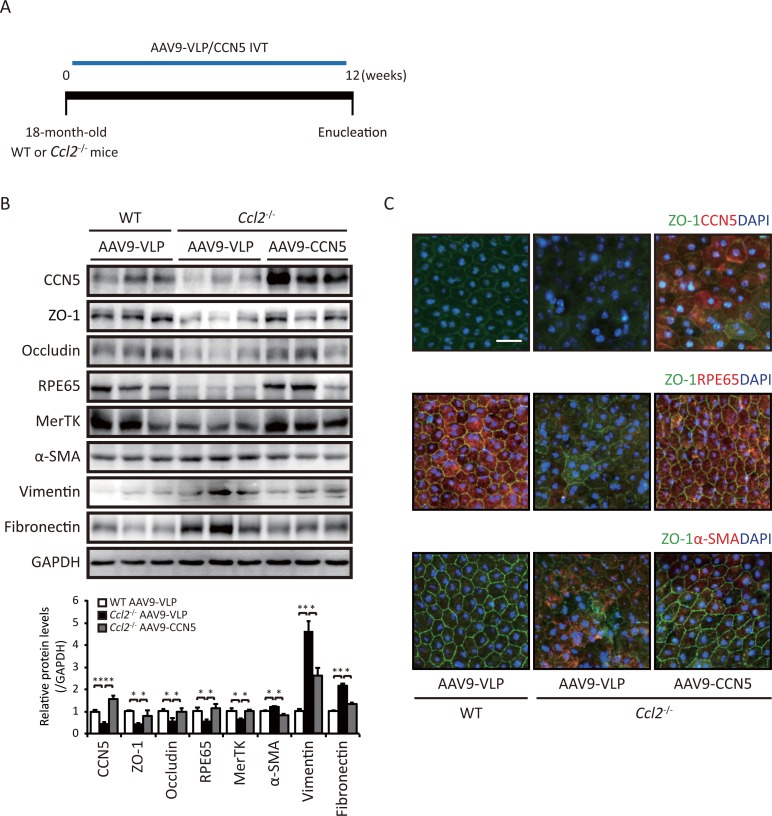
CCN5 is protective in a mouse model of AMD. (A) The effects of intravitreally injected AAV9-CCN5 in 18-month-old *Ccl2*^-/-^ mice were evaluated at 12 weeks post-injection. Viral particles corresponding to 2 × 10^9^ viral genomes in 2 μl were used per eye. (B) Western blot analysis of RPE/choroid/sclera complex lysates isolated from 18-month-old *Ccl2*^-/-^ mice and age-matched WT littermates using antibodies against CCN5, α-SMA, vimentin, fibronectin, ZO-1, occludin, RPE65, and MerTK. GAPDH was used as a loading control. Quantified protein levels were normalized against the loading control (left bottom) (n = 4, WT AAV9-VLP; n = 6, *Ccl2*^-/-^ AAV9-VLP; and n = 6, *Ccl2*^-/-^ AAV9-CCN5). Error bars = S.D. *p < 0.05, **p < 0.01. (C) RPE flat mounts were co-immunostained against ZO-1 (green) and CCN5 (red) or RPE65 (red) or α-SMA (red). Using DAPI for nucleus staining (blue). The representative images are at least three independent experiments. Scale bar: 150 μm. Original magnifications: 200×.

Aged *Ccl2*^-/-^ mice exhibit a subset of phenotypes of dry-type AMD, including fibrotic deformation of RPE [24]. A recombinant adeno-associated virus (AAV) expressing CCN5 (AAV9-CCN5) [20] or a control virus (AAV9-VLP) was intravitreally injected into 18-month-old *Ccl2*^-/-^ mice. RPE cells were obtained and analyzed at 12 weeks post-injection (Fig 5A). The CCN5 level was markedly reduced in *Ccl2*^-/-^ mice but was restored by AAV9-CCN5. Levels of the mesenchymal marker proteins (α-SMA, vimentin, and fibronectin) were significantly increased in RPE of *Ccl2*^-/-^ mice, whereas levels of the tight junction marker proteins (ZO-1 and occludin) and of the functional RPE marker proteins (RPE65 and MerTK) were significantly decreased, and AAV9-CCN5 normalized these alterations (Fig 5B). To investigate the integrity of tight junction, RPE/choroid/sclera complex were mounted. Compared to the organized hexagonal shape of tight junction in the AAV9-VLP delivered 18-month-old wild-type (WT) littermate mice, age-matched AAV9-VLP delivered *Ccl2*^-/-^ mice showed irregular and vague shape of tight junction. In contrast, tight junction integrity was maintained in *Ccl2*^-/-^ mice under the intravitreal injection of AAV9-CCN5 as assessed by immunocytochemical staining of ZO-1 (Fig 5C). The RPE65 level was markedly reduced and the α-SMA level was prominently elevated in *Ccl2*^-/-^ mice, which were significantly normalized by the injection of AAV9-CCN5 (Fig 5C). These data suggest that RPE undergoes fibrotic deformation in aged *Ccl2*^-/-^ mice and that this can be reversed by restoration of the CCN5 level.

## Discussion

Phenotypic transformation of RPE has been implicated in various ocular diseases. This change is often described as epithelial-mesenchymal transition (EMT), which leads to loss of epithelial characteristics, including down-regulation of tight junction proteins, and gain of mesenchymal phenotypes, including up-regulation of α-SMA, vimentin, and fibronectin. However, more comprehensive cellular and molecular analyses are required to clarify the TGF-β-mediated phenotypic transformation of RPE. We thus called this phenomenon “fibrotic deformation” throughout this study. TGF-β can induce fibrotic deformation of RPE *in vitro* [25–27]. This effect is of physiological relevance. For example, TGF-β reactivity has been detected in vitreous samples, subretinal fluids, and epiretinal membranes surgically removed from patients with retinal detachment [28].

In this study, we report that CCN5 is expressed in RPE, as detected by western blotting (Fig 1 and S1 Fig) and qRT-PCR (S3 Fig), although the role of CCN5 in normal RPE is unknown. Intriguingly, the CCN5 level was markedly reduced in TGF-β-treated RPE cells, and restoration of the CCN5 level prevented TGF-β-induced deformation of RPE cells (Figs 1 and 2, S1–S3 Figs). As a control, an antagonist of TGF-β receptor, A83-01, was also treated along with TGF-β to RPE cells. Western blotting showed that the effects of CCN5 and A83-01 in preventing the TGF-β-induced malformation of RPE cells were comparable (S4 Fig). The TGF-β-SMAD signaling pathway was significantly inhibited by CCN5 as assessed by western blotting (S5 Fig). These observations are consistent with our previous findings that CCN5 inhibits the TGF-β-SMAD signaling pathway in the heart [19, 20] by up-regulating inhibitory SMAD7 (unpublished observations). It was also shown that CCN5 is strongly expressed in non-invasive breast cancer cells in which it down-regulates the expression of TGF-β receptor II (TGF-βRII) at the transcriptional level. Loss of CCN5 promoted EMT of the breast cancer cells through up-regulation of the TGF-βRII level [29]. Therefore, the inhibitory role of CCN5 in the TGF-β-SMAD signaling appears to be conserved in diverse cells and tissues.

CCN5 also antagonizes the pro-fibrotic activity of CCN2 (also known as connective tissue growth factor), although the underlying mechanism is unknown [19]. CCN2 was significantly up-regulated in RPE cells treated with TGF-β, and this change was prevented by CCN5 (S4 and S5 Figs). Treatment with a combination of EGTA, epidermal growth factor, and fibroblast growth factor was suggested to be a more physiologically relevant means of inducing fibrotic deformation of RPE [30]. CCN5 prevented RPE deformation induced by this combination of reagents (data not shown). Collectively, these data suggest that CCN5 is required to maintain the epithelial phenotypes of RPE and that restoration of the CCN5 level prevents fibrotic deformation of RPE induced by diverse pathological stimuli.

More clinically important is the finding that CCN5 reversed pre-established fibrotic deformation of RPE, as shown by the reappearance of tight junction proteins and the disappearance of mesenchymal marker proteins (Fig 3). This phenomenon is reminiscent of mesenchymal-epithelial transition (MET). Notably, CCN5 enhances MET of pancreatic cancer cells [31] and loss of CCN5 promotes EMT and acquisition of stem cell-like phenotypes in estrogen-dependent MCF7 breast cancer cells [32]. Therefore, the role of CCN5 in MET-like phenotypic transformation is not confined to RPE. In this regard, it is of note that the ARPE-19 cell line spontaneously acquired immortality [33] and thus is not truly representative of native RPE cells. Further analyses of primary cultures of RPE cells are needed to confirm these findings and to elucidate the underlying signaling pathways.

We extended our findings from *in vitro* experiments to an *in vivo* model of fibrotic ocular disease. *Ccl2*^-/-^ mice have morphological, ultrastructural, and functional features characteristic of AMD. Substantial Bruch’s membrane thickening and drusen deposition are observed in aged *Ccl2*^-/-^ mice. Lipofuscin granules, which are thought to promote RPE dysfunction in dry-type AMD, accumulate in RPE of 15-month-old *Ccl2*^-/-^ mice. In addition, attenuation of RPE and chorio-capillaries are observed in 16-month-old *Ccl2*^-/-^ mice [24]. We focused on the pathogenic alteration of RPE cells in *Ccl2*^-/-^ mice. The CCN5 level was significantly reduced concomitant with apparent EMT-like fibrotic deformation of RPE in 18-month-old *Ccl2*^-/-^ mice, and this was rescued by restoration of the CCN5 level, as assessed by western blotting and immunohistochemistry (Fig 5). Although extensive functional and anatomical analyses were not performed, our data show that CCN5 induced phenotypic reversion of deformed RPE cells *in vivo*.

Taken together, our data suggest that CCN5 is a novel modality to prevent and reverse fibrotic deformation of RPE cells.

## Supporting information

S1 Fig Fibrotic conformation of ARPE-19 cells was induced by TGF-β. ARPE-19 cells were cultured for 48 hours with 5 ng/ml or 10 ng/ml of TGF-β. (A) Compared with control, TGF-β treatment cells were transformed to mesenchymal morphology by bright field microscopy. The representative images are at least three independent experiments. Original magnifications: 100×. Scale bar: 200 μm. (B) CCN5 and tight junction markers are down-regulated, whereas mesenchymal markers are up-regulated during TGF-β-induced fibroblast-like phenotype in ARPE-19 cells. Error bars = S.D. *p<0.05, **p<0.01.
(EPS)

S2 Fig TGF-β induces fibrotic changes in ARPE-19 cells. Cells were visualized by staining with anti-α-SMA and phalloidin. The representative images are at least three independent experiments. Original magnification: 200×. Scale bar: 50 μm.
(EPS)

S3 Fig CCN5 maintains mRNA levels of tight junction markers and attenuates mesenchymal markers from TGF-β-induced fibrotic changes of ARPE-19 cells. Quantitative RT-PCR analyses of CCN5, ZO-1, occludin, RPE65, α-SMA, vimentin, and fibronectin (A, B, and C) were performed (n = 4). Error bars = S.D. *p<0.05, **p<0.01.
(EPS)

S4 Fig An antagonist of TGF-β receptor, A83-01, prevents TGF-β-induced fibroblast-like RPE disruption. ARPE-19 cells were pretreated with 1 μM of A83-01 for 1 hour and cultured with TGF-β for 48 hours. Cell lysates were subjected to Western blotting. Error bars = S.D. *p<0.05, **p<0.01.
(EPS)

S5 Fig CCN5 inhibits TGF-β-SMAD signaling pathway. ARPE-19 cells were cultured in the presence of AdLacZ or AdCCN5 prior to TGF-β treatment. Cell lysates were subjected to Western blotting. Error bars = S.D. *p<0.05, **p<0.01.
(EPS)

S1 Table List of primary antibodies. (DOCX)

S2 Table Sequences of primers used for qRT-PCR. (DOCX)
