# Supplementary material for: The matricellular protein CCN5 inhibits fibrotic deformation of retinal pigment epithelium
Source: PLoS One. 2018 Dec 20;13(12):e0208897. doi: 10.1371/journal.pone.0208897 (PMC6301692; doi:10.1371/journal.pone.0208897)
Supplement: S1 Table — (DOCX) [file pone.0208897.s006.docx]

**Supplementary table 1. List of primary antibodies**

| **Antibody** | **Company** | **Dilution** | **Applications** | **Source** |
| --- | --- | --- | --- | --- |
| ZO-1 | Invitrogen | 1:1000, 1:200 | WB/ICC, IHC | Rabbit |
| ZO-1 | Invitrogen | 1:200 | IHC | Mouse |
| Occludin | Invitrogen | 1:1000 | WB | Rabbit |
| Fibronectin | Sigma-Aldrich | 1:1000 | WB | Rabbit |
| α-SMA | Sigma-Aldrich | 1:1000, 1:200 | WB/ICC, IHC | Mouse |
| Vimentin | Santa Cruz | 1:1000 | WB | Mouse |
| HA | Roche | 1:1000 | WB | Mouse |
| Type I collagen | Abcam | 1:1000 | WB | Mouse |
| CCN5 | GenScript | 1:1000 | WB | Mouse |
| CCN5 | OriGene | 1:200 | IHC | Rabbit |
| MerTK | R&D systems | 1:1000 | WB | Goat |
| RPE65 | Abcam | 1:1000, 1:200 | WB, IHC | Mouse |
| TGF-βRII | Santa Cruz | 1:1000 | WB | Mouse |
| SNAI1 | Santa Cruz | 1:1000 | WB | Mouse |
| SNAI2 | Santa Cruz | 1:1000 | WB | Mouse |
| pSMAD2 | Cell Signaling Technology | 1:1000 | WB | Rabbit |
| SMAD2/3 | Cell Signaling Technology | 1:1000 | WB | Rabbit |
| SMAD4 | Cell Signaling Technology | 1:1000 | WB | Rabbit |
| SMAD7 | Cell Signaling Technology | 1:1000 | WB | Rabbit |
| CCN2 | Santa Cruz | 1:1000 | WB | Goat |
| GAPDH | Abcam | 1:1000 | WB | Rabbit |
