# Supplementary material for: The matricellular protein CCN5 inhibits fibrotic deformation of retinal pigment epithelium
Source: PLoS One. 2018 Dec 20;13(12):e0208897. doi: 10.1371/journal.pone.0208897 (PMC6301692; doi:10.1371/journal.pone.0208897)
Supplement: S2 Table — (DOCX) [file pone.0208897.s007.docx]

**Supplementary table 2. Primer sequences for quantitative real-time PCR**

| **Gene** | **Sequence** |
| --- | --- |
| 18S rRNA | 5′- GTG GAG CGA TTT GTC TGG TT -3′  5′- CGC TGA GCC AGT CAG TGT AG -3′ |
| ZO-1 | 5′- GAT GAT CGT CTG TCC TAC CTG TC -3′  5′- GAG TCC TCT CTT ACA GGC TCA GA -3′ |
| Occludin | 5′- GAA GCC AAA CCT CTG TGA G -3′  5′- GAA GAC ATC GTC TGG GGT GT -3′ |
| RPE65 | 5′- ATG GAC TTG GCT TGA ATC ACT T -3′  5′- GAA CAG TCC ATG AAA GGT GAC A -3′ |
| α-SMA | 5′- CCT CAC AGA GAG AGG CTA TTC CT -3′  5′- GCA GCT CAT AGC TCT TCT CCA G -3′ |
| Fibronectin | 5′- CTG GCC GAA AAT ACA TTG TAA A -3′  5′- CCA CAG TCG GGT CAG GAG -3′ |
| Vimentin | 5′- GAG AAC TTT GCC GTT GAA GC -3′  5′- CGT GAT GCT GAG AAG TTT CG -3′ |
| CCN5 | 5′- GTC CCC TTT CCT CTA ACT CAC TG -3′  5′- GTG TGC CTT CTC TTC ATC CTA CC -3′ |
